# Aptamer blocking S-TLR4 interaction selectively inhibits SARS-CoV-2 induced inflammation

**DOI:** 10.1038/s41392-022-00968-2

**Published:** 2022-04-11

**Authors:** Gang Yang, Shengnan Zhang, Yuchun Wang, Ling Li, Yu Li, Deyu Yuan, Fatao Luo, Jincun Zhao, Xu Song, Yongyun Zhao

**Affiliations:** 1 Center for Functional Genomics and Bioinformatics, College of Life Science, Sichuan University, Chengdu, Sichuan 610064 P.R. China; 2 State Key Laboratory of Respiratory Disease, Guangzhou Institute of Respiratory Disease, The First Affiliated Hospital of Guangzhou Medical University, Guangzhou, Guangdong 510182 China

**Keywords:** Infectious diseases, Target identification

**Dear Editor**,

Excessive inflammatory responses lead to increased mortality from the severe acute respiratory syndrome coronavirus 2 (SARS-CoV-2). Nearly all deceased patients infected with SARS-CoV-2 are found to have cytokine storm syndrome and viral sepsis. They are prone to superinfections exacerbating the course of disease.^1^ Therefore, preventing hyperinflammation is critical for avoiding this cytokine storm syndrome. Currently, specific immunomodulators are under clinical research or application, including targeting inflammatory cytokines (sarilumab, anakinra, tocilizumab, infliximab, adalimumab) and inflammatory pathway (baricitinib, ruxolitinib).^2^ However, they may also increase the risk of superinfections.^3^ Therefore, it is urgent to develop highly selective anti-inflammatory drugs which don’t cause superinfections to effective treatment of SARS-CoV-2.

The role of innate immune receptors in the response to SARS-CoV-2 has been explored. It was demonstrated that the spike protein of SARS-CoV-2 (S), a trimeric protein and highly glycosylated class I fusion protein, is capable of interacting with and activating TLR4 to induce inflammatory cytokines (IL-1B and IL-6) production.^4^ Therefore, specifically blocking the S-TLR4 interaction to inhibit inflammatory responses should be a novel therapeutic protocol. Recently, several DNA aptamers have been reported with neutralization activity against SARS-CoV-2 by blocking the interaction of the RBD domain of spike protein and ACE2.^5^ Nevertheless, aptamers that can inhibit inflammatory responses have not been developed. Hence, we screened spike protein against DNA aptamers, aiming to selectively inhibit inflammation caused by SARS-CoV-2.

After 12 rounds of selection, aptamer ST-6 clearly stands out from other candidate sequences due to its high inhibition of blocking the S-TLR4 interaction (Supplementary Fig. S1a–e). Interestingly, aptamer ST-6 is Guanine(G)-rich sequences and can be assembled into G-quadruplexes (Fig. 1a and Supplementary Fig. S1f, g). According to this structure, we truncated and optimized this aptamer, and obtained two truncated aptamers (ST-6-1 and ST-6-2) (Supplementary Table S1). Disruption of the G-quadruplex significantly reduces the binding ability (Supplementary Fig. S1h). Subsequently, their characteristics were analyzed. The dissociation constants (*K*_D_) of those three aptamers were determined to be in the range of 36 nM to 80 nM (Fig. 1b). Consistently, the results of flow cytometry and fluorescent images also demonstrate that those aptamers have high binding affinity (Fig. 1c and Supplementary Fig. S2a). Additionally, they bind only to spike proteins of SARS-CoV-2, neither spike proteins of other viruses (SARS-CoV, MERS, HCoV-HKU1) nor other proteins (IgG, PDGF-AA, ACE2, TLR4, BSA, RBD) (Supplementary Fig. S2b). This indicates that these aptamers have ideal selectivity, reducing the potential for off-target cytotoxicity. Furthermore, they also showed high stability and low immunogenicity in mice (Supplementary Fig. S2c–e). Overall, those characteristics show that those aptamers have good pharmaceutical potential.

**Fig. 1:**
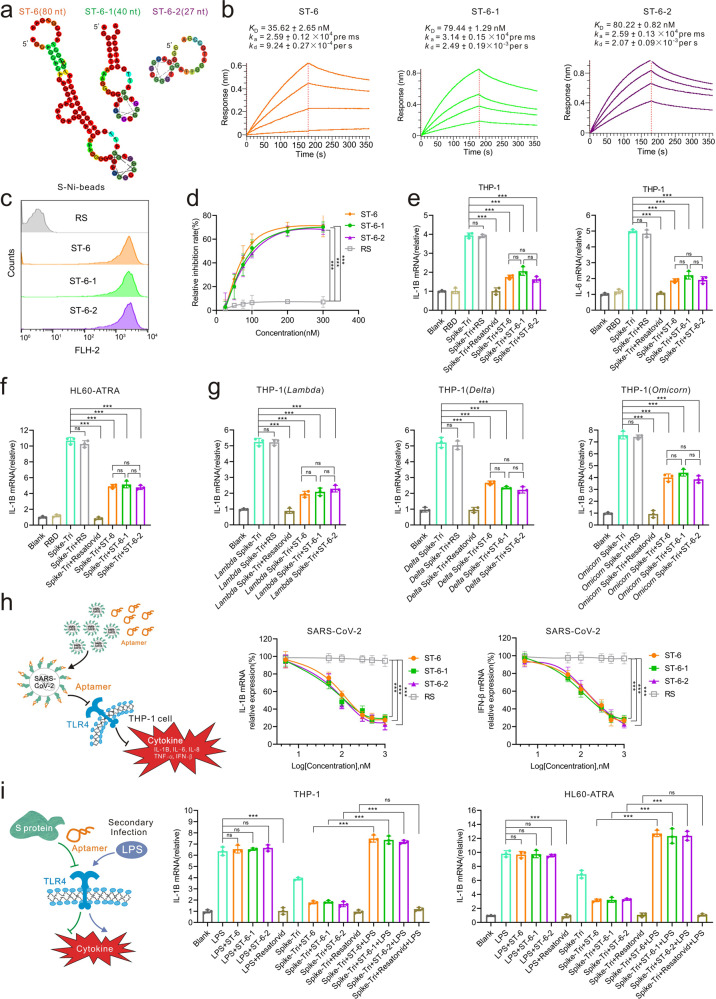
Aptamers selectively inhibit SARS-CoV-2 induced inflammation. **a** The secondary structure of the aptamer ST-6 (orange), ST-6-1 (green) and ST-6-2 (purple) were predicted using RNAfold by adjusting the DNA and G-quadruplex parameter. **b** BLI assay for the kinetic binding parameters of aptamer binding to immobilized spike proteins. **c** Flow cytometry to investigate the binding performance of cy3-labeled aptamer (200 nM) to spike protein (2 μg) pre-bind to Ni-beads (3 μL). Random sequences (RS) were used as baseline controls. **d** ELISA reveals the efficiency of aptamers in prevention experiments. Aptamer (200 nM) was incubated with spike protein (100 ng/well) pre-coated on a microplate before the addition of TLR4 (200 ng/well). **e**, **f** qRT-PCR analysis for the expression of cytokine in cells treated with 10 nM SARS-Spike-Tri or RBD of spike protein, 10 nM SARS-Spike-Tri + 200 nM aptamer, 10 nM SARS-Spike-Tri + 100 μM Resatorvid for 2 h. SARS-Spike-Tri is short for the spike protein trimer of SARS-CoV2. RS: Random sequences. **g** The inhibition efficiency of aptamer on IL-1B production in THP-1 cells stimulated with spike protein from SARS-CoV-2 variants (the Delta, Lambda and Omicron variants). THP-1 cells were treated with 10 nM SARS-Spike-Tri-variant, 10 nM SARS-Spike-Tri- variant + 200 nM aptamer, 10 nM SARS-Spike-Tri- variant + 100 μM Resatorvid for 2 h. SARS-Spike-Tri-variant is short for the spike protein trimer of SARS-CoV2 variants. RS: Random sequences. **h** qRT-PCR analysis for the cytokine inhibition in THP-1 cells infected with 2 × 10^6^ PFU/mL authentic SARS-CoV-2 with different concentrations of aptamers for 2 h. RS: Random sequences. **i** qRT-PCR analysis of immune response to LPS in cells pretreated with spike-Tri and aptamer. Cells treated with 50 ng/mL LPS, 50 ng/mL LPS + 200 nM aptamer, 50 ng/mL LPS + 100 μM Resatorvid, 10 nM SARS-Spike-Tri, 10 nM SARS-Spike-Tri + 200 nM aptamer, 10 nM SARS-Spike-Tri + 100 μM Resatorvid, 10 nM SARS-Spike-Tri + 200 nM aptamer, 10 nM SARS-Spike-Tri + 200 nM aptamer + 50 ng/mL LPS, 10 nM SARS-Spike-Tri + 100 μM Resatorvid + 50 ng/mL LPS for 2 h. All the error bars indicate standard deviations (*n* = 3). All the *P* values were determined using paired *t* test. ***P < 0.001.

We evaluated the blocking capacity of those aptamers. In the prevention experiment with simulated SARS-CoV-2 prevention reagents, those aptamers replaced about 70% TLR4 bound to the spike protein (Fig. 1d). Moreover, to simulate the window period and infected patients, those aptamers also showed significant blockade in competition and substitution experiments, respectively (Supplementary Fig. S3a, b). Additionally, to explore the structural basis for aptamer blocking the S-TLR4 interaction, molecular dynamics simulations showed that the aptamer and TLR4 were bound to the same epitope of spike protein, and TLR4 didn’t interact with RBD (Supplementary Fig. S3c, d). Furthermore, since the aptamer doesn’t bind to RBD, it doesn’t block the S-ACE2 interaction (Supplementary Fig. S3e, f). Those have verified the details of the aptamer suppressing S-TLR4 interaction. Taken together, those aptamers showed their potential therapeutic properties.

To evaluate the anti-inflammatory potential, monocytes and neutrophils cells were stimulated by spike proteins, with or without pre-bound aptamer, respectively. Resatorvid, a selective TLR4 inhibitor that inhibits cytokines production, was used as the positive control. As expected, those three aptamers remarkably reduce cytokines (IL-1B, IL-6, TNF-α and IFN-β) production in THP-1 cells (a cell line of human monocytes) (Fig. 1e and Supplementary Fig. S4a, b). We also treated HL-60 cells (a promyelocytic leukemia cell line) with all-trans retinoic acid (ATRA) which directed HL-60 cells to differentiate into neutrophils. Likewise, the same results were observed in ATRA-differentiated HL-60 cells (Fig. 1f and Supplementary Fig. S4c). More interestingly, they also reduce IL-1B production in THP-1 cells which are stimulated by spike proteins of SARS-CoV-2 variants, including the Delta, Lambda and Omicron (Fig. 1g and Supplementary Fig. S4d–f), demonstrating that those aptamers exert ideal universality and robust anti-inflammatory potency. Subsequently, aptamers were assessed for inhibition of authentic SARS-CoV-2 induced inflammation. They inhibit cytokines (IL-1B, IFN-β, IL-6, IL-8, TNF-α) production in a dose-dependent manner. The IC_50_ is estimated to be in the range of 47.89 to 65.52 nM and they reduced the amounts of cytokines by approximately 75 %, indicating that those aptamers displayed a high anti-inflammatory potency against authentic SARS-CoV-2 virus (Fig. 1h and Supplementary Fig. S4g).

A major challenge in designing anti-inflammatory agents is to balance efficacy and safety, especially to ensure that it doesn’t affect the host’s defense against other bacterial or viral infections. Hence, we wondered whether those aptamers could impair the host’s immune function. Lipopolysaccharide (LPS) stimulated immune responses are mediated by Toll-like receptors on the surface of host cells was used to simulate secondary infection (Fig. 1i). There is no cytokine production in THP-1 cells pretreated with Resatorvid and spike proteins, suggesting that TLR4 inhibitors impair the normal immune function of the host. Nevertheless, fortunately, THP-1 cells pretreated with aptamers and spike proteins reproduced cytokines under LPS stimulation, indicating that the TLR4 signal transduction pathway maintained normal immune responses. Consistently, this phenomenon is also observed in ATRA-differentiated HL-60 cells. Overall, those results demonstrate that those three aptamers are highly selective inhibitors and don’t impair the host’s immune function to cause superinfection.

In summary, three aptamers that effectively prevent inflammation by specifically blocking S-TLR4 interaction are developed. They remarkably reduced the release of cytokines in monocytes and neutrophils, regardless of whether the stimulus came from spike proteins of wild-type or SARS-CoV-2 variants. They also exhibited robust anti-inflammatory potential against authentic SARS-CoV-2. Notably, due to their high selectivity and low immunogenicity, the possibility of these aptamers causing superinfection is very low. Therefore, these three DNA aptamers will provide highly selective anti-inflammatory agents without causing superinfection and may reduce inflammation symptoms before they become severe. Combining these aptamers with antiviral infection drugs may be used as a potential treatment against SARS-CoV-2.

## Supplementary information

Supplementary Materials

## Data Availability

All data are available from the corresponding author.

## References

[1] Huang C, Wang Y, Li X (2020). Clinical features of patients infected with 2019 novel coronavirus in Wuhan, China. Lancet.

[2] Henderson LA (2020). On the alert for cytokine storm: immunopathology in COVID-19. Arthritis Rheumatol..

[3] Rizk JG (2020). Pharmaco-immunomodulatory therapy in COVID-19. Drugs.

[4] Zhao Y (2021). SARS-CoV-2 spike protein interacts with and activates TLR41. Cell Res..

[5] Yang G (2021). Identification of SARS-CoV-2-against aptamer with high neutralization activity by blocking the RBD domain of spike protein 1. Signal Transduct. Target Ther.

