# Supplementary material for: Aptamer blocking S-TLR4 interaction selectively inhibits SARS-CoV-2 induced inflammation
Source: Signal Transduct Target Ther. 2022 Apr 11;7:120. doi: 10.1038/s41392-022-00968-2 (PMC8996214; doi:10.1038/s41392-022-00968-2)
Supplement: Supplementary file 1 — Supplementary Materials [file 41392_2022_968_MOESM1_ESM.docx]

Supplementary Materials for
**Aptamer Blocking S-TLR4 Interaction Selectively Inhibits SARS-CoV-2
Induced Inflammation**

Gang Yang ^1, †^, Shengnan Zhang ^1, †^, Yuchun Wang ^1, †^, Ling Li ^1, †^, Yu Li^1^, Deyu Yuan^1^, Fatao Luo^1^, Jincun Zhao^2,^ *, Xu Song^1,^ *, Yongyun Zhao^1,^ *

^1^ Center for Functional Genomics and Bioinformatics, College of Life Science, Sichuan
University, Chengdu, Sichuan 610064, P.R. China.
^2^ State Key Laboratory of Respiratory Disease, Guangzhou Institute of Respiratory Disease, The
First Affiliated Hospital of Guangzhou Medical University, Guangzhou, Guangdong, 510182,
China.

^†^ These authors contributed equally.
^*^ Correspondence to: Yongyun Zhao (yongyunfly@163.com), Xu Song (xusong@scu.edu.cn),
Jincun Zhao ([zhaojincun@gird.cn](mailto:zhaojincun@gird.cn)).

**This PDF file includes:**Materials and Methods
Figures. S1 to S4
Tables S1
Reference

**Materials and Methods**

**Materials**

His-tagged Spike trimer recombinant protein of SARS-CoV-2 was purchased from novoprotein Inc. (DRA49, Shanghai, China). The Spike trimer recombinant protein of the His-tagged SARS-CoV-2 lambda variant was purchased from ACRO Biosystems Inc. (SPN-C52Hs, USA). The Spike-trimer recombinant protein of the His-tagged SARS-CoV-2 Delta variant was purchased from novoprotein Inc. (DRA168, Shanghai, China). TLR4 protein was purchased from Sino Biological Inc. (10146-H08B, Beijing, China). TLR4 polyclonal antibody was purchased from Proteintech Inc. (19811-1-AP, USA). HisPur^TM^ Cobalt Resin (Co-beads) was purchased from Thermo Scientific. Inc. (VB291130, USA). His-tag protein pure Ni-Beads (Ni-Beads) were purchased from BioMag Beads (BMNI-5, Wuxi, China). SARS-CoV Spike trimer recombinant protein, MERS Spike trimer recombinant protein and HCoV-HKU1 spike trimer recombinant protein were purchased from ACRO Biosystems Inc. (SPN-M52H4, SPN-S52H6, SPN-H52H5, USA). Dip and Read^TM^ Biosensors streptavidin (SA) were purchased from ForteBio (California, USA). All media for cell culture were purchased from Gibco (USA). Fetal bovine serum (FBS) was purchased from Excell Bio (Shanghai, China) and penicillin-streptomycin was purchased from Hyclone (USA). All types of DNA sequences with HPLC purification were synthesized by Sangon Biotech (Shanghai, China).

**SELEX procedures**

His-tagged SARS-CoV-2 Spike-trimer protein was used as the target and His-tag protein was used as the negative control. An ssDNA library (5′-AGCAGCACAGAGGTCAGATG-[N]40-CCTATGCGTGCTACCGTGAA-3′ (N = A, T, G, or C)) consisting of 40 nt random core sequence and constant 20 nt arm sequences at both ends were used for target-based SELEX. Briefly, the synthetic ssDNA library was incubated with His-tagged S proteins conjugated to HisPurTM Cobalt Resin (S-Co-beads) for SELEX enrichment. S-Co-beads were incubated with the library at 25°C for 30 min in the binding buffer contained 1×PBS buffer with magnesium ions (10 mM Na2HPO4, 2 mM KH2PO4, 137 mM NaCl, 2.7 mM KCl, 0.55 mM MgCl2, pH=7.4), and then separated and washed twice with the washing buffer (PBST: the binding buffer with 0.05 % Tween 20). Subsequently, the bound oligonucleotides were eluted by Milli-Q H2O at 80℃ for 7 min with mild shaking. The selected ssDNA was amplified by PCR. To convert dsDNA to ssDNA, the Lambda exonuclease enzyme was applied to separate and remove the 5′-phosphorylated antisense sequences from the sense sequences. Then, the ssDNA purified using NucleoSpin® Extract II kit was subjected for the next round of SELEX. After 3 rounds, the enriched ssDNA sequences were firstly subjected to counter-selection with control His-tag proteins conjugated to HisPurTM Cobalt Resin (Co-beads) and combined enrichment. To increase the selection pressure1, after 6 rounds, the amount of Spike protein was decreased from 2 μg to 1 μg. After 9 rounds, the amount of Spike protein was decreased from 1 μg to 500 ng, 2 μg TLR4 protein was simultaneously added to elute the bound oligonucleotides, and the supernatant was collected for amplification. After 12 rounds of selection, the enriched libraries were subjected to high-throughput sequencing (Sangon Biotech, Shanghai, China)

**Competition blocking assay of S-TLR4 interaction**

96-well ELISA Microplates (446469, Thermo Scientific^TM^ Immuno, USA) were pre-coated with S proteins (8 nM) in 100 μL coating buffer (pH=9.6, 100 mM NaHCO_3_) at 4 ℃ overnight. After washing two times, 200 μL blocking solution (2 % BSA in PBST) was added at room temperature (RT) for 1 h with mild shaking. For blocking purposes, serial dilutions of aptamers (25, 50, 75, 100, 200, 300 nM) were added into microplates and incubated at RT for 30 min. Meanwhile, the blocking solution, random sequences and ssDNA library were used as baseline control, respectively. Then, TLR4 (30 nM) was added into microplates and incubated at RT for 30 min with gentle shaking. Microplates were washed twice to remove free TLR4 and aptamers. To detect the remaining amount of TLR4, TLR4 polyclonal antibody (1:1,000 diluted in the blocking solution) was added to the microplates and incubated at RT for 1 h. After washing three times, HRP-conjugated goat anti-rabbit IgG antibody (1:1,000 diluted in the blocking solution) was added into microplates. After washing three times, the 3,3′,5,5′-Tetramethylbenzidine (TMB) substrate was added to the microplates (100 μL per well) and incubated for 15 min at RT. The reaction was stopped by the addition of 2 M H_2_SO_4_ (50 μL per well) and the absorbance was measured at 450 nm using a microplate reader.

**Flow cytometry analysis**

To evaluate the binding performance of selected aptamers, S proteins (40 nM) coated in Ni-beads were incubated with 200 nM cy3-labeled candidate sequences in 100 μL binding buffer at RT for 30 min. Meanwhile, Ni-beads incubated with cy3-labeled random sequences were used as the negative control. The beads were washed twice using washing buffer and suspended in 1 mL binding buffer. The fluorescence intensity of beads with counting about 5000 events was measured by flow cytometry[^2^](#_ENREF_2) (FACSVerse, BD). Meanwhile, the data was analyzed using FlowJo (V10, BD).

**Binding assays** **of an aptamer to protein**

SARS-CoV-2 S proteins (100 ng per well) were coated in the 96-well ELISA Microplates with 100 μL coating buffer at 4 °C overnight. The S-protein-coated plates were washed twice with washing buffer before blocking with 200 μL of the solution (2% BSA in PBST) at RT for 1 h. Then, 200 nM final concentration biotin-labeled aptamers at the 5′-end were added into the wells and incubated in binding buffer at RT for 30 min with gentle shaking. After washing two times, streptavidin-horseradish peroxidase (HRP) and its substrates were sequentially added into the reactions. Color development was carried out using 3,3′,5,5′- Tetramethylbenzidine (TMB) substrate and measured using a microplate reader at 450 nm absorbance.

**Fluorescence microscope imaging**

Spike protein Ni-beads (5 μL beads and about 2 μg spike protein) or negative Ni-beads were incubated with 200 nM FAM-labeled candidate sequences in 100 μL binding buffer at RT for 30 min. After washing two times using washing buffer, the beads were suspended in 100 μL binding buffer and the fluorescence was monitored by fluorescence microscope (Leica DMi 8, USA). The fluorescence intensity was quantified by Image J software (2021, USA).

**Bio-layer interferometry (BLI)**

Streptavidin biosensors were pre-wetting in the PBST/Mg^2+^ for 10 min. Then, the sensors were immersed into other 96-well added 200 μL PBST/Mg^2+^ (0.55 mM) and shaking (1000 rpm, same as the following steps) for 90 s (baseline phase). For loading purposes, streptavidin biosensors were used to capture biotinylated aptamers (the thickness signal was 0.6 nm) in PBST/Mg with shaking for 10 min (loading phase). After washing in the PBST/Mg^2+^ for 60 s (second baseline phase), the loaded sensors were immersed into a serial diluted of S proteins (25, 50, 75, 100 nM) with shaking for 3 min (association phase). Then, the sensors were immersed into PBST/Mg^2+^ for an additional 3 min (disassociation phase). The background signal was measured using a reference sensor with biotinylated aptamers loading but without S protein binding and subtracted from the corresponding S protein binding sensor. Binding affinity kinetic features using ForteBio Octet K2 (Pall, USA). A 1:1 binding model and the ForteBio data analysis software were used for curve fit. Average *k*_a_, *k*_d_, *K*_D_ values were measured by averaging all binding curves that matched the theoretical fit with an R^2^ value of 0.98.

**Molecular docking and dynamic simulations**

The structure of proteins (SARS-CoV-2 S protein, TLR4/MD-2 heterodimer) were obtained from the RCSB PDB data bank (http://www.rcsb.org, ID: 6VYB, ID: 3VQ1). These aptamers have Guanine(G)-rich sequences and seem they can assemble into G-quadruplexes formed[^3^](#_ENREF_3)^,^[^4^](#_ENREF_4). We adjusted the DNA and G-quadruplex parameters to predict the secondary structure of those aptamers by RNAfold server. The predicted G4 motif was selected for its structure prediction using the 3D-Nus server. The extra 5’ and 3’ nucleotide bases were added using Discovery Studio v4.0 (DS).

Molecular docking was performed with HDOCK after obtaining the 3D structures of aptamers and their target. The complex structure between the S protein and the S-aptamer molecule was predicted using a hybrid protein-DNA docking algorithm, HDOCK. Specifically, given the individual structure of the protein and the S-aptamer molecule, HDOCK used a fast Fourier transform (FFT)-based search strategy to globally sample all possible binding modes between the two molecules. Then, all the sampled binding modes were evaluated by the iterative knowledge-based scoring function ITScorePP. Last, the binding modes were ranked according to their binding energy scores, and the top ten binding modes were provided. During the docking calculation, all the default parameters were used. The binding residues analysis was performed by the HDOCK webserver. In addition, the binding free energy of the protein-aptamer complex was calculated by MD simulations. Finally, docking molecules were visualized using VMD. Also, binding residues are labeled.

**Fluorescence single-wavelength measurements**

The experiment was performed as described[^5^](#_ENREF_5)^,^[^6^](#_ENREF_6), using a 96-well microplate from Greiner (Flat Bottom Black Polystyrol). Oligonucleotides (1 μM) and ThT (3 μM) (T3516-5G, Sigma, USA) were mixed, respectively. Samples were incubated in 50 mM Tris-HCl, pH 7.2, and 50 mM KCl with a volume of 100 μL. Fluorescence emission was collected at 492 nm after excitation at 412 nm in a microplate reader (Infinite M1000Pro, Tecan). Each condition was tested at least in triplicate for each DNA sample. Measurements were performed at room temperature.

**Circular dichroism (CD) experiments**

CD experiments were carried out on DNA samples at a concentration of 10 μM in 400 μL 1×PBS buffers[^5^](#_ENREF_5) (10 mM Na_2_HPO_4_, 2 mM KH_2_PO_4,_ 137 mM NaCl, 2.7 mM KCl, pH=7.4). CD studies were carried out on a JASCO J-815 spectrometer (Japan) attached with a peltier temperature controller (model: PTC-423S). The spectra were measured in the wavelength range 200-600 nm using quartz cuvettes with a path length of 1.0 mm. The scanning speed of the instrument was 100 nm/min, and 2 s was the response time.

**Agarose gel electrophoresis analysis of aptamer stability**

1 μg aptamer was added to the binding buffer containing 10 % FBS and incubated at 37 °C for different times (0, 3, 6, 12, 24 and 48 h). The random sequence was incubated at 37 °C for 12 h. Then, the DNA samples were loaded onto 2 % agarose gel in 1×TAE buffer and run at 120 V for 30 min. Gels were incubated in Ethidium bromide (1 μg/ml) for 5 min and bands were imaged using Gel Doc XR + Gel imaging system (Bio-rad, Hercules, California, USA) with ultraviolet excitation (302 nm).

**Cell culture**

THP-1 cells were purchased from FuHeng BioLogy (FH0112, Shanghai, China) and HL-60 cells (CCL-240) were purchased from American Type Culture Collection (ATCC, USA). THP-1 cells and HL-60 cells were cultured in RPMI 1640 medium (Gibco) supplemented with 100 U/mL Penicillin-Streptomycin and 10 % (v/v) fetal bovine serum (Gibco), in a humidified atmosphere containing 5% CO_2_ incubator at 37 °C. Cells were negative for mycoplasma.

**HL-60 cells differentiation**

HL-60 cells were differentiated according to the previous research[^7^](#_ENREF_7). HL-60 cells were passaged every 3 days and no more than 15 passages were used in all experiments. The cells were incubated with ATRA (1 μmol/L) for 5 days. HL-60 cells differentiate into granulocyte-like cells. Then, cells were collected into a 15 mL tube. And cells naturally precipitated for 2 h and were resuspended by RPMI 1640 medium with 10% FBS. Differentiated cells were counted before the experiment.

**Aptamers inhibit inflammatory responses in immune cells**

The bioactivity of S protein has been proved by ELISA assay (binding to TLR4). THP-1 cell and HL-60 cell were used to evaluate the ability of aptamers to inhibit inflammatory responses. Initially, S protein (10 nM) was mixed with a serial of diluted aptamers in binding buffer at RT for 10 min. Subsequently, the mixtures of S and aptamers were added to the immune cells and incubated at 37 °C for 2 h. After stimulation, cells were collected and centrifuged at 3000 rpm for 5 min at 4 °C. Subsequently, trizol was used to extract RNA. qRT-PCR was used to detect the inflammatory cytokine including IL-1β, IL-6, TNF-α, IFN-β. In addition, random sequences were used as the negative control, and resatorvid was used as the positive control of aptamers.

Authentic SARS-CoV-2 virus (2×10^6^ PFU/mL) were mixed with serially diluted aptamers (5, 50, 100, 300, 500, 1000 nM) in binding buffer at RT for 10 min. Then, 4×10^5^ of THP-1 cells were incubated with mixtures of SARS-CoV-2 and aptamers at 37 °C for 2 h. After incubating, THP-1 cells were harvested to detect the inflammatory cytokine (IL-1β, IL-6, IL-8, TNF-α, IFN-β). All SARS-CoV-2 infection experiments were performed in Guangzhou Gustoms District Technology Center Biosafety Level 3 (BSL-3) Laboratory.

**Quantitative RT-PCR (qRT-PCR) analysis**

Total RNA was isolated from the stimulated cells by RNAiso Plus (108-95-2, TaKaRa, Japan). Then, cDNA was prepared using DNase I (01056834, Thermo Scientific, USA) and RevertAid Reverse Transcriptase (00991337, Thermo Scientific, USA). qRT-PCR was performed using the Applied Biosystems 7500 Real-Time PCR Systems (Thermo Fisher Scientific, USA) with 2×Real PCR Easy^TM^ Mix-SYBR (210520, Foregene, China). The data of qRT-PCR were analyzed by the Livak method (2-ΔΔCt). ACTB was used as a reference gene for the human cell lines.

**Mice**

Female C57BL/6 and BALB/c mice (6-8-weeks old) were provided by Laboratory Animal Center of Sichuan University. All mice were kept in sterile, autoclaved cages and provided with enough food and water. All animal experiments were undertaken at Sichuan University and approved by the local regulatory agency.

**Stability of aptamers in mice**

Cy5.5-labeled aptamers or random-sequence oligonucleotides (1.6 mg/kg) in 150 μL physiological saline solution were intraperitoneally administrated to mice (C57BL/6). The ﬂuorescent signals were detected by Xenogen IVIS Imaging System 200 Series (Caliper Life Sciences, Alameda) at the indicated time. In the quantitative analysis, the average value of the fluorescence signal emitted by the buffer-treated group was defined as the autofluorescence background signal

**Aptamers induce cytokine responses in mice**

For *in vivo* stimulation, 200 μL physiological saline solution containing aptamers was incubated for 20 mins. Subsequently, mice (BALB/c) were randomly separated into five groups and injected through the tail vein. The injection amount of the aptamer is 30 mg/kg. Control mice received either physiological saline solution (blank) alone or LPS (3 mg/kg) (positive control), respectively. Whole blood samples were obtained by tail clippings at the indicated time. Then, serum was prepared from whole blood by coagulation at 37 °C for 30 min and centrifugation. Mouse cytokine (IL-1B, IL-6, TNF-α) was measured using a mouse cytokine (IL-1B, IL-6, TNF-α) Valukine^TM^ ELISA (Novus Biologicals, the minimum detectable dose in pg/ml) according to the product information and manual.

**Statistical analysis**

All analyses were repeated at least three times, and a representative experimental result was presented. Data were analyzed using GraphPad Prism version 8.0 (GraphPad Software, San Diego, CA). Continuous variables with normal distribution are expressed as the mean ± standard deviation (SD). Comparisons between groups were all verified for normal distribution by D’Agostino-Pearson omnibus test. Student’s t-test (for pairwise comparisons) and one-way ANOVA (for comparisons among three or more groups) were used. The post hoc test with Bonferroni correction was performed for multiple comparisons following ANOVA.

Figures S1

**
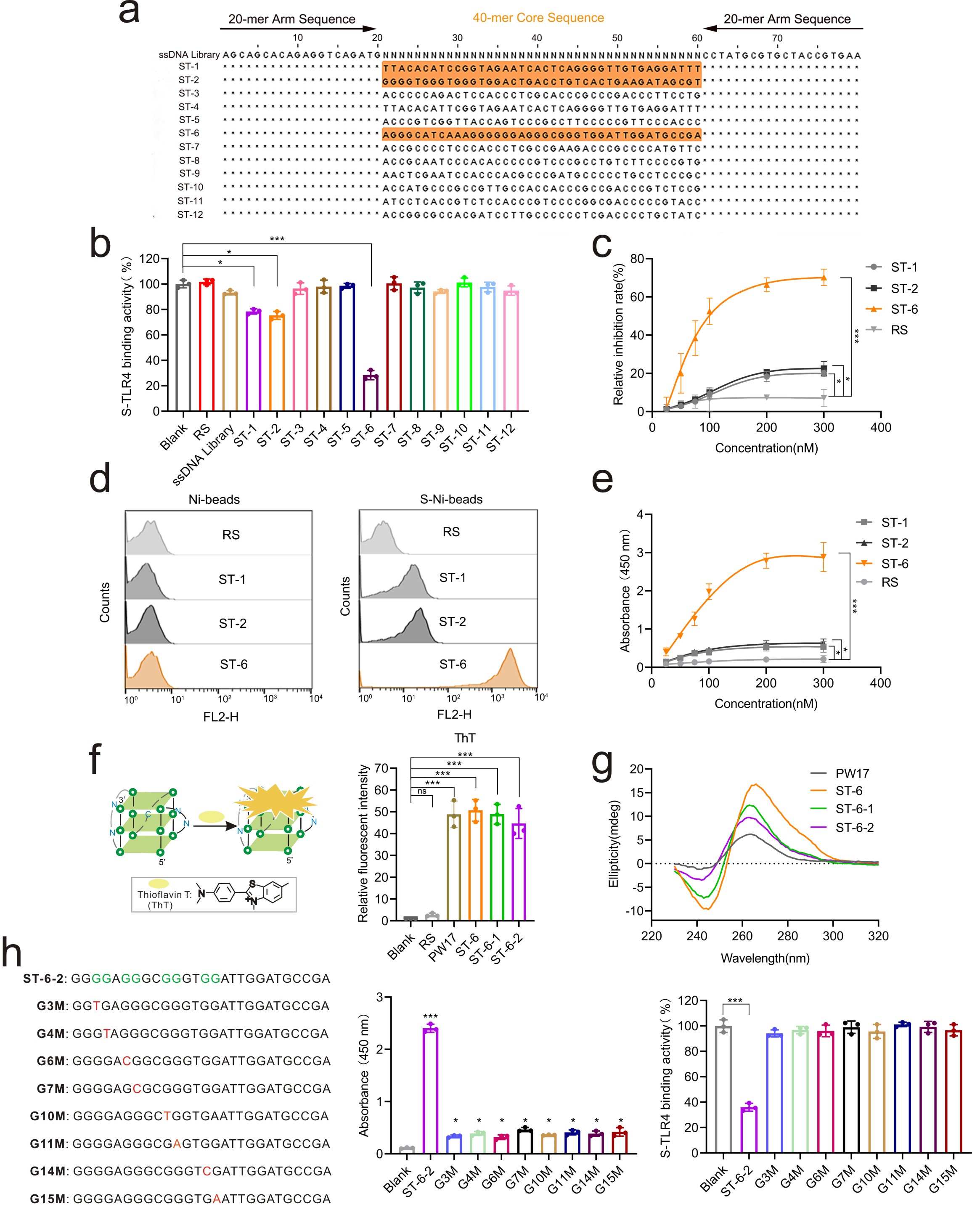
**

**Supplementary Fig. S1.** **Identification of aptamers**. **a**. The sequence of 12 candidate sequences. (ST-1—12). **b.** Competition blocking assay to evaluate the efficiency of the candidate sequences (200 nM) that prevents TLR4 (200 ng/well) binding to spike protein (100 ng/well) pre-coated on microplates. **c**. The inhibition of aptamers (ST-1, ST-2 and ST-6) at different concentrations to prevent TLR4 (200 ng/well) binding to spike proteins (100 ng/well). **d**. Flow cytometry to investigate the binding performance of cy3-labeled candidate aptamer (ST-1, ST-2 and ST-6) (200 nM) to spike protein (2 μg) pre-bind to Ni-beads (3 μL). Random sequences were used as baseline controls. Ni-beads were used as the negative control. **e**. Binding assays to assess the binding performance of biotin-labeled aptamer (200 nM) to spike protein (100 ng/well) pre-coated on microplates. **f**. The schematic diagram of G-quadruplex binding to Thioflavin T (ThT) to produce fluorescence and the fluorescence intensity of ThT binding to aptamer ST-1, ST-2 and ST-6, respectively. PW17 was taken as the positive control which adopt G4 structure. **g**.CD spectra recorded for aptamer ST-1, ST-2 and ST-6 in 1 × PBS buffer, respectively. The structure of those three aptamers fold into parallel quadruplexes (CD peak at 260 nm, valley at 240 nm), respectively. **h**. Binding assays to assess the binding performance of biotin-labeled mutated sequences (200 nM) to spike protein (100 ng/well) pre-coated on microplates and ELISA reveals the inhibition of the mutated aptamers to prevent TLR4 binding to spike proteins pre-coated on microplates. Aptamer (200 nM) was incubated with spike protein (100 ng/well) pre-coated on a microplate before the addition of TLR4 (200 ng/well). The mutated base is labeled in red and the bases corresponding to ST-6-2 are marked in green. All the error bars indicate standard deviations (n= 3). All the *P* values were determined using paired t-test. **P*＜0.05; ****P*＜0.001.

Figures S2


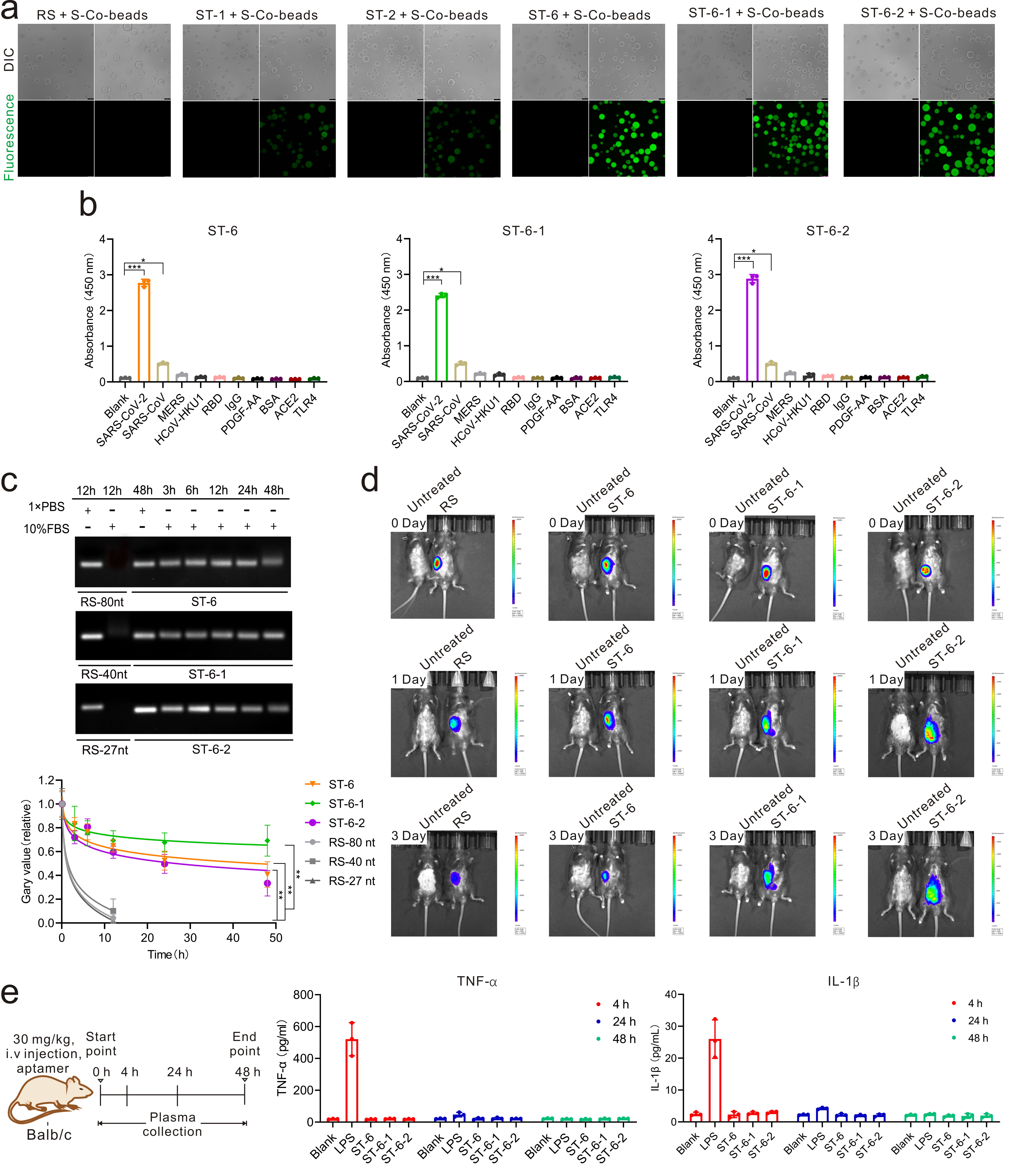


**Supplementary Fig. S2. The characteristics of** **aptamers ST-1, ST-2 and ST-6. a**. Fluorescence images to monitor the binding performance of FAM-labeled candidate sequences (200 nM) to 100 μL binding buffer containing spike-Co-beads (5 μl beads and 2 μg Spike protein) and its fluorescence statistics. RS was used as the negative control. **b**. Selectivity study of those three aptamers against the spike protein of coronaviruses (SARS-CoV2, SARS-CoV, MERS-CoV, HCoV-HKU1) and other proteins (IgG, PDGF-AA, ACE2, TLR4 and BSA). RBD: the receptor-binding domain (RBD) of spike protein. **c**. Stability analysis of those three aptamers incubated in 10% FBS at 37 ℃ for different times and the quantitative analysis. **d**. The stability of Cy5.5-labeled aptamers (1.6 mg/kg) after a single subcutaneous injection in mice was analyzed by whole-body fluorescence imaging. Cy5.5-labeled RS was used as the negative control. **e**. The immunogenicity of those three aptamers in mice (N= 3) was evaluated by ELISA. Those three aptamers were dissolved in physiological saline solution and administered intravenously (i.v.) in the tail vein of Balb/c mice, respectively. Serum samples were collected at 4 h, 24 h and 48 h after injection, and the levels of inflammatory cytokines were measured. The amount of the aptamer injected intravenously into the mice is 30 mg/kg. Blank is for 0 h. All the error bars indicate standard deviations (n= 3). All the *P* values were determined using paired t-test. **P*＜0.05; ****P*＜0.001.

Figures S3

**
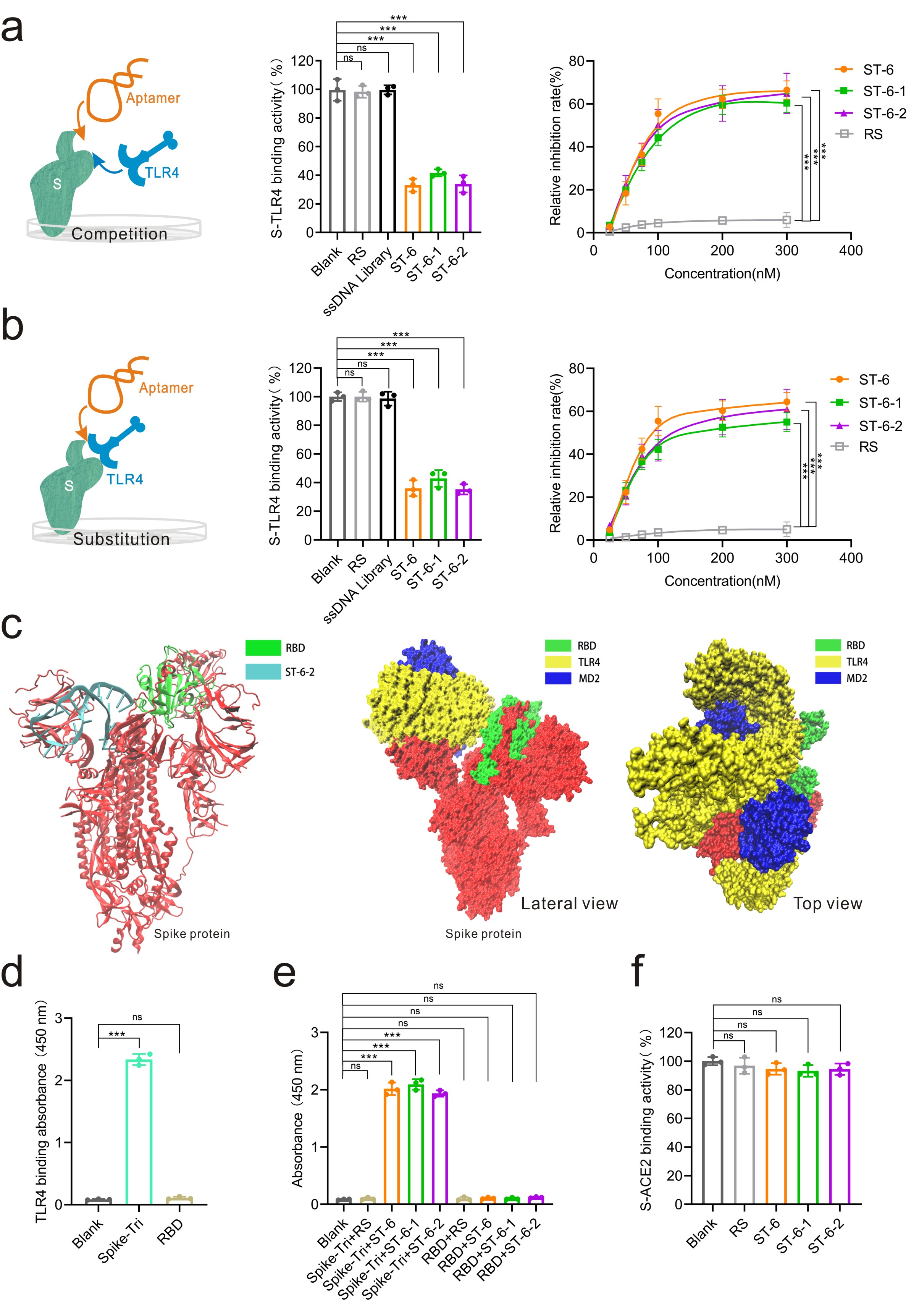
**

**Supplementary Fig. S3. The blocking capacity of aptamers ST-1, ST-2 and ST-6. a.** Schematic illustration and TLR4 inhibition efficiency of aptamers on competition and ELISA show the competitive inhibition of TLR4 binding to spike proteins by aptamers. In the competition experiment, spike protein (100 ng/well), TLR4 (200 ng/well) and the aptamer (200 nM) were mixed at the same time to simulate the window period of SARS-CoV-2 infection. **b**. Schematic illustration of the inhibition of aptamers on substitution and ELISA reveals the efficiency of aptamers to substitute TLR4 binding to spike proteins. Aptamer (200 nM) was added into the solution of the spike protein (100 ng/well) pre-bound to TLR4 (200 ng/well) to simulated an infected patient. **c**. The epitope comparison between ST-6 and TLR4. Docking analysis of ST-6-2-spike protein complex and TLR4-spike protein complex. TLR4 and MD-2 form a heterodimer that recognizes spike proteins. RBD: the receptor-binding domain (RBD) of spike protein. **d**. ELISA reveals that RBD does not interact with TLR4. TLR4 (100 ng/well) was incubated with RBD (100 ng/well) pre-coated on a microplate. The S-TLR4 interaction was used as the positive control. **e**. Binding assays to assess the binding performance of biotin-labeled aptamer (ST-1, ST-2 and ST-6, 200 nM) to RBD (100 ng/well) pre-coated on microplates. Random sequences (RS) were used as baseline control. Spike protein was used as the positive control. **f**. ELISA reveals the inhibition of those three aptamers to prevent ACE2 binding to spike proteins pre-coated on microplates. Aptamer (200 nM) was incubated with spike protein (100 ng/well) pre-coated on a microplate before the addition of ACE2 (200 ng/well). All the error bars indicate standard deviations (n= 3). All the *P* values were determined using paired t-test. **P*＜0.05; ****P*＜0.001.

Figures S4

**
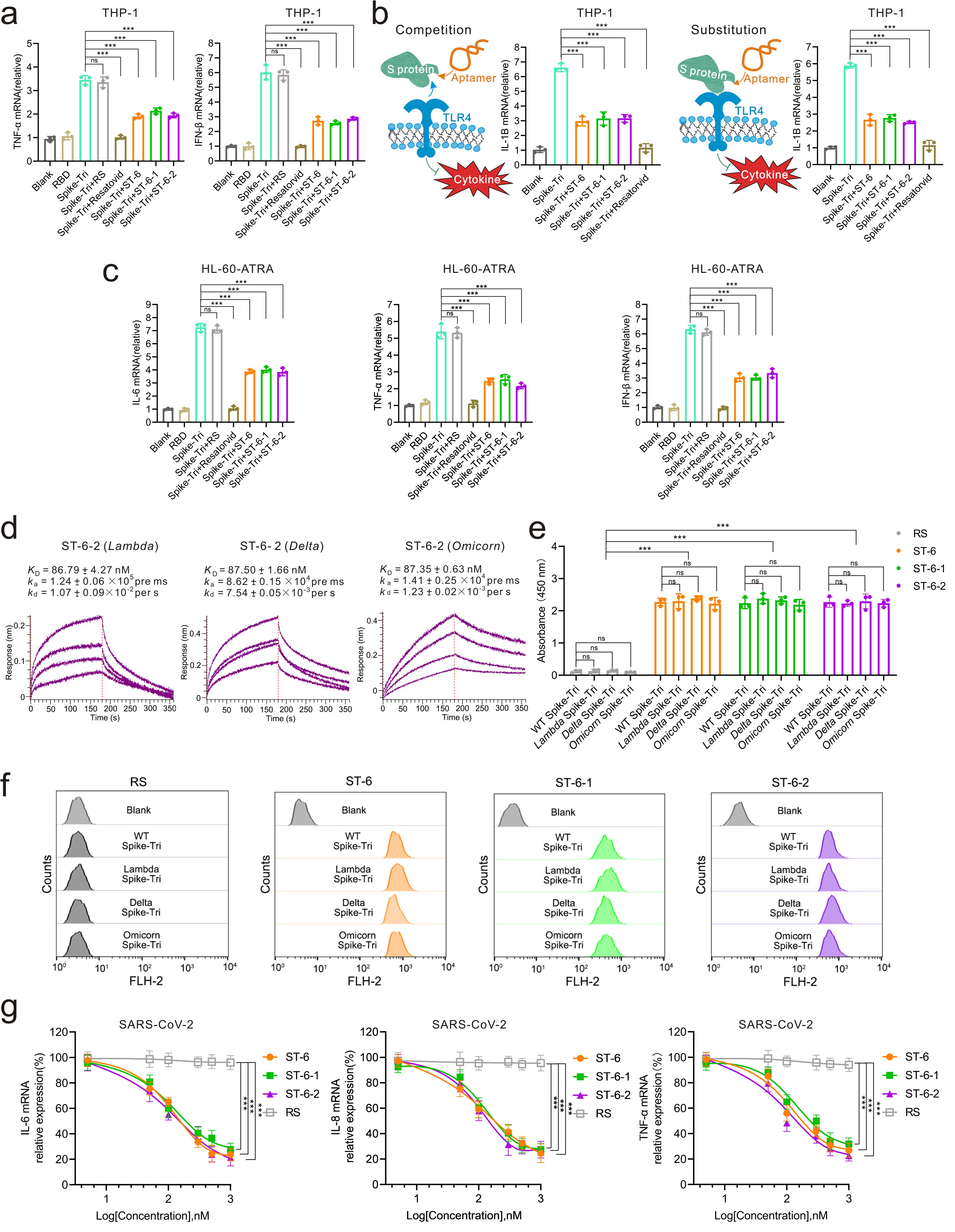
**

**Supplementary Fig. S4. The anti-inflammatory potential of aptamers ST-1, ST-2 and ST-6**. **a.** qRT-PCR analysis for the expression of cytokines (TNF-α, IFN-β) in THP-1 cells treated with 10 nM SARS-Spike-Tri or RBD of spike protein, 10 nM SARS-Spike-Tri + 200 nM aptamer (ST-6, ST-6-1, ST-6-2) or random sequence (RS), 10 nM SARS-Spike-Tri + 100 μM Resatorvid for 2 h. Spike-Tri is short for the spike protein trimer of SARS-CoV2. RBD was used as the negative control of spike protein. RS was used as the negative control of aptamer and Resatorvid was used as the positive control. **b**. IL-1B expression in THP-1 cells was analyzed by qRT-PCR in competitive test of simulated infection window and substitute test of simulated infected patients, respectively. **c**. qRT-PCR analysis for the expression of cytokines (IL-6, TNF-α, IFN-β) in ATRA (1 μM)-differentiated HL-60 with different treatments. **d**. BLI assay for the kinetic binding parameters of aptamer ST-6-2 binding to immobilized spike proteins from SARS-CoV-2 variants (the Delta, Lambda and Omicron variants). **e**. Binding assays to assess the binding performance of biotin-labeled aptamer (ST-1, ST-2 and ST-6, 200 nM) to spike protein from SARS-CoV-2 variants (the Delta, Lambda and Omicron variants, 100 ng/well) pre-coated on microplates. Random sequences (RS) were used as baseline controls. WT was used as the positive control. **f**. Flow cytometry to investigate the binding performance of cy3-labeled aptamer (ST-1, ST-2 and ST-6, 200 nM) to spike protein from SARS-CoV-2 variants (the Delta, Lambda and Omicron variants, 2 μg) pre-bind to Ni-beads (3 μL). Random sequences (RS) were used as baseline controls. WT was used as the positive control. Blank: without protein. **g**. qRT-PCR analysis for the cytokines (IL-6, IL-8, TNF-α) inhibition in THP-1 cells infected with 2×10^6^ PFU/mL authentic SARS-CoV-2 with different concentrations of aptamers (ST-6, ST-6-1, ST-6-2) for 2 h. All the error bars indicate standard deviations (n= 3). All the *P* values were determined using paired t-test. **P*＜0.05; ****P*＜0.001.

**Supplementary** **Tables S1**

Table S1. The information of the DNA sequence used in the experiment.

| **Name** | **Sequences (5'-3')** |
| --- | --- |
|  |  |
| ST-6  (80 nt) | AGCAGCACAGAGGTCAGATGAGGGCATCAAAGGGGGGAGGGCGGGTGGATTGGATGCCGACCTATGCGTGCTACCGTGAA |
| ST-6-1  (40 nt) | AGGGCATCAAAGGGGGGAGGGCGGGTGGATTGGATGCCGA |
| ST-6-2  (27 nt) | GGGGAGGGCGGGTGGATTGGATGCCGA |
| ST-1 | AGCAGCACAGAGGTCAGATGTTACACATCCGGTAGAATCACTCAGGGGTTGTGAGGATTTCCTATGCGTGCTACCGTGAA |
| ST-2 | AGCAGCACAGAGGTCAGATGGGGGTGGGTGGGTGGACTGACCTGTCACTGAAGATAGCGTCCTATGCGTGCTACCGTGAA |
| IL-1B | TGATGGCTTATTACAGTGGCAATGAGGATGAC |
|  | CCTTGCTGTAGTGGTGGTCGGAGATTCG |
| IL-6 | ACAAGCGCCTTCGGTCCAGTTGCCTTCT |
|  | TTCGTTCTGAAGAGGTGAGTGGCTGTCT |
| IL-8 | ATGACTTCCAAGCTGGCCGTG |
|  | AATTCTCAGCCCTCTTCAAAAACTT |
| TNF-α | GACGTGGAACTGGCAGAAGAG |
|  | TCGCACAAGCAGGAATGAGA |
| IFN-β | ATGAGCACTGAAAGCATGATC |
|  | TCACAGGGCAATGATCCCAAAGT |
| Rs | TAGATCAGTCCTAATAGACCATTCGAATCGAGCTATGATTAAGCTAGACTTGAAGATAC |
| RS-80 nt | GCTGTGTGACTCCTGCAACCACCGACTCGCCGCACCACCGCCAATTCACGTGTCCATCGCAGCTGTATCTTGTCTCCAAT |
| RS-40 nt | CGAGAATTCAAAAAGCGTGGACTGTACCCTCCACTTTTAT |
| RS-27 nt | AACACCGGTGCCATCAGTCGCCGGTCC |

**Reference:**

1 Sun, M. *et al.* Aptamer Blocking Strategy Inhibits SARS-CoV-2 Virus Infection. *Angew. Chem.Int. Edit*. **60**, 10266-10272, (2021).

2 Zheng, G. *et al.* A genetically encoded fluorescent biosensor for monitoring ATP in living cells with heterobifunctional aptamers. *Biosens. Bioelectron*. **198**, (2022).

3 Chen, J.N. *et al.* Regulation of PDGFR-beta gene expression by targeting the G-vacancy bearing G-quadruplex in promoter. *Nucleic Acids Res*. **49**, 12634-12643, (2021).

4 Li, X. m. *et al.* Guanine-vacancy-bearing G-quadruplexes responsive to guanine derivatives. *P. Nat. Acad. Sci.* **112**, 14581-14586, (2015).

5 Mohanty, J. *et al.* Thioflavin T as an Efficient Inducer and Selective Fluorescent Sensor for the Human Telomeric G-Quadruplex DNA. *J. Am. Chem. Soc*. **135**, 367-376, (2013).

6 de la Faverie, A. R. *et al.* Thioflavin T as a fluorescence light-up probe for G4 formation. *Nucleic Acids Res*. **42**, (2014).

7 Zhao, Y. *et al.* SARS-CoV-2 spike protein interacts with and activates TLR41. *Cell Res*. **31**, 818-820, (2021).
